# Image steganalysis using LSTM fused convolutional neural networks for secure telemedicine

**DOI:** 10.3389/fmed.2025.1619706

**Published:** 2025-09-01

**Authors:** Doaa Shehab, Mohmmed Alhaddad

**Affiliations:** Faculty of Computing and Information Technology, King Abdulaziz University, Jeddah, Saudi Arabia

**Keywords:** steganalysis, steganography, data hiding, healthcare security, LSTM, lightweight

## Abstract

Deep learning-based image steganalysis has progressed in recent times, with efforts more concerted toward prioritizing detection accuracy over lightweight frameworks. In the context of AI-driven health solutions, ensuring the security and integrity of medical images is imperative. This study introduces a novel approach that leverages the correlation between local image features using a CNN fused Long Short-Term Memory (LSTM) model for enhanced feature extraction. By replacing the fully connected layers of conventional CNN architectures with LSTM, our proposed method prioritizes high-relevance features, making it a viable choice for detecting hidden data within medical and sensitive imaging datasets. The LSTM layers in our hybrid model demonstrate better sensitivity characteristics for ensuring privacy in AI-driven diagnostics and telemedicine. Experiments were conducted on Break Our Steganographic System (BOSS Base 1.01) and Break Our Watermarking System (BOWS) datasets, followed by validation on the ALASKA2 Image Steganalysis dataset. The results confirm that our approach generalizes effectively and would serve as impetus to ensure security and privacy for digital healthcare solutions.

## 1 Introduction

AI-based digital healthcare solutions require security and data privacy while handling sensitive medical images; therefore, robust techniques are essential to maintain data integrity (1, 2). Particularly, the medical images contain embedded metadata and annotations that may compromise patient privacy (3). Image steganalysis helps in preserving sensitive medical records (4) and by leveraging artificial intelligence (AI) techniques, healthcare professionals can identify potential threats posed by steganographic attacks (5, 6). Beyond privacy concerns, the integrity of medical data is another essential dimension for AI diagnostic systems (7, 8). Malicious actors could use steganography to manipulate images, alter tumor regions, or embed misleading data without detection (1). Advanced steganalysis techniques and emerging telemedicine issues necessitate the integration of robust AI-driven steganalysis tools to improve the security of sensitive health data (2).

Recent image steganalysis techniques exploited the traditional machine learning to extract meaningful features, but human dependencies limited their scope in image steganalysis (9). Low embedding capacity and poor image retrieval rates necessitated the deployment of deep learning assisted steganalysis algorithms. Detailed reviews regarding the recent deep learning strategies and network developments are included elsewhere (10, 11). In this connection, numerous deep learning algorithms were reported for rapid detection of steganographic payloads with reasonable accuracies (12–15). Key modifications include enhancing filters and different activation operators (16), high-order co-occurrence matrices to capture sensitivity (17, 18), periodic weight capture (19), dimensionality reduction schemes (20), and covariance pooling techniques (16, 21–24).

Moreover, various DL-based models such as Qian et al. (25), Yedroudj et al. (18), Boroumand et al. (19), Deng et al. (16), Zhang et al. (26), Reinel et al. (22), Öztürk Ş and Özkaya (27), and Ozdemir et al. (28) tried to improvise on the stego image feature extraction. In this regard, You et al. (29) exploited EfficientNet, MixNet, and ResNet by removing pooling and stride operations in the first layers. Similarly, (24) applied floating-point quantization to XuNet (24). Recently, LSTM was reported to capture data correlation for image classification tasks (21, 30–32).

In this study, we propose a CNN architecture fused with LSTM by replacing the fully connected layers of the CNN. Our proposed model leverages LSTM to optimize weight matrices and bias vector parameters, ensuring effective training at each time step. In addition, LSTM nodes extract essential contextual features, which is vital for detecting hidden threats within medical images. This research contributes to the field by demonstrating the effectiveness of LSTM fused CNNs in medical image steganalysis by offering a robust security framework to protect sensitive patient data. Furthermore, we compare our proposed architecture with state-of-the-art deep learning models in terms of computational efficiency. By significantly reducing the number of trainable parameters, our model offers a resource efficient and scalable solution for secure medical image transmission and integrity in telemedicine.

The remaining of this work is organized as follows: Explain the Architecture of CNN and LSTM in Section 2. The materials and methods are presented in Section 3. The results discussion is detailed in Section 4. Section 5 concludes the study.

## 2 A brief on CNN and LSTM architecture

**Figure 1:**
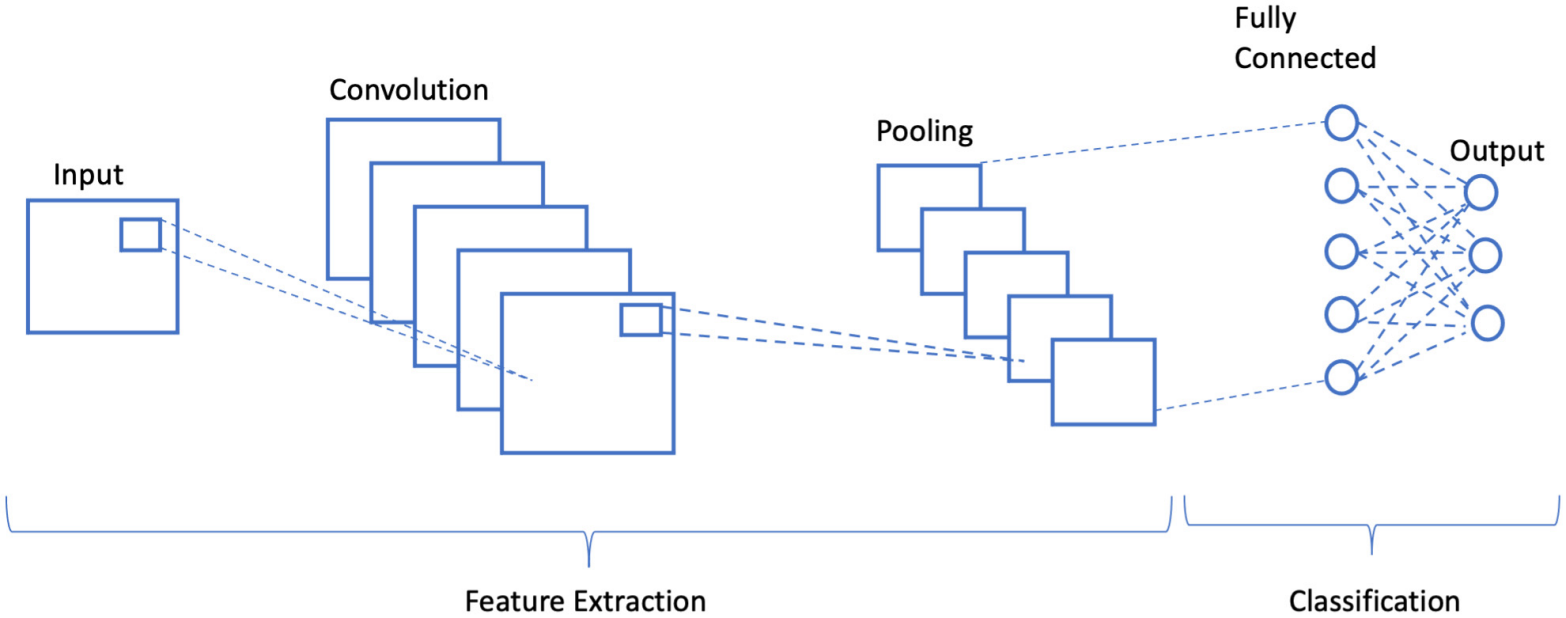
Schematic illustration of a generalized convolution neural network.

The encoder in any CNN-based steganography scheme employs binary inputs: one for the cover image and the other for secret image to foster a stego image. It includes pre-processing, feature extraction, and classification stage as illustrated in Figure 1. In the feature extraction phase, convolution is performed multiple times to ameliorate the signal-to-noise ratio of the image and to characterize local features, whereas in classification, the extracted local features are average-pooled and concatenated to yield final feature maps. These feature maps were then classified in terms of class probabilities using SoftMax function.

**Figure 2:**
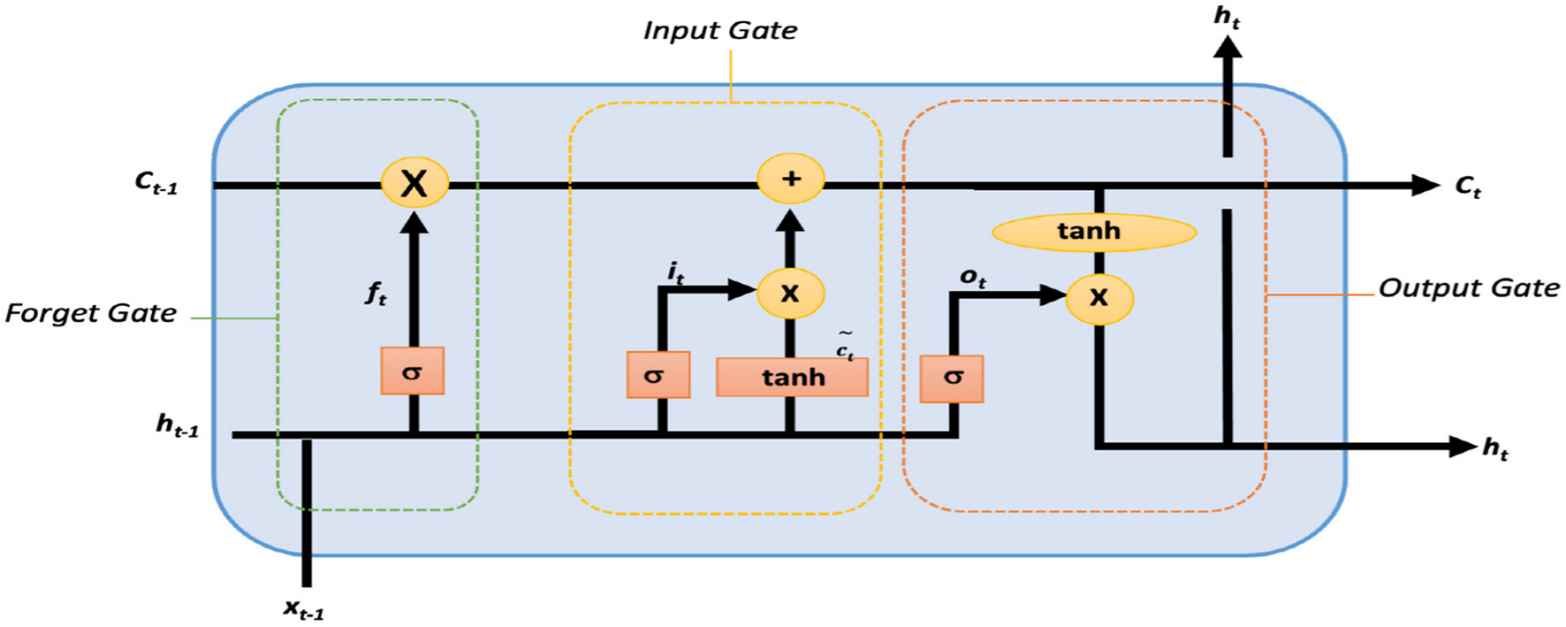
Internal architecture of a single LSTM cell.

Though LSTM networks improve the functioning of recurrent neural networks (RNNs) in terms of vanishing gradient, LSTM contains three gates which are an input gate, a forget gate, and an output gate, where *x*_*t*_, *C*_*t*_, and *C*_*t*−1_ represent the current input, new, and previous cell states, respectively. *h*_*t*_ and *h*_*t*−1_ refer to the current and previous outputs, respectively. A non-linear function is used to activate these three gates, which makes LSTM a dynamic model with changing contexts (33). The internal architecture of an LSTM cell is shown in Figure 2.

Within an LSTM cell, forget gate controls the contribution of the previous state *C*_*t*−1_ to the current state by using sigmoid function σ and is responsible for LSTM cell memory as given by the expression in Equation 1.


(1) $$\begin{array}{l} f_{t}=\sigma \left(W_{f}\cdot \left[h_{t-1},x_{t}\right]+b_{f}\right) \end{array}$$

where *f*_*t*_ is the forget vector, and *x*_*t*_ and *h*_*t*−1_ are the current input and previous output. As given in Equation 1, *x*_*t*_ and *h*_*t*−1_ are multiplied by the trained weights matrix *W*_*f*_ with offset *b*_*f*_. Due to sigmoid function, the input vector ranges between 0 and 1, indicating the degree to which values are to be remembered or forgotten. *h*_*t*−1_ and *x*_*t*_ are passed via input updated gate to append the relevant information and is governed by Equation 2. Thereafter, new information is obtained as $\overset{\sim }{C_{t}}$ from Equation 3 after passing *h*_*t*−1_ and *x*_*t*_ via tanh function. Finally, the candidate of the cell state *C*_*t*_ for the next time step is generated by combining current moment information $\overset{\sim }{C_{t}}$ and long-term memory information *C*_*t*−1_ as shown in Equation 4.


(2) $$\begin{array}{l} i_{t}=\sigma \left(W_{i}\cdot \left[h_{t-1},x_{t}\right]+b_{i}\right) \end{array}$$

(3) $$\begin{array}{l} \overset{\sim }{C_{t}}=\tanh\left(W_{i}\cdot \left[h_{t-1},x_{t}\right]+b_{i}\right) \end{array}$$

(4) $$\begin{array}{l} C_{t}=f_{t}C_{t-1}+i_{t}\overset{\sim }{C_{t}} \end{array}$$

Here, *W*_*i*_ denotes weight matrices that are produced from sigmoid function, and *b*_*i*_ denotes the input gate bias. The output gate controls the require output *O*_*t*_ using the expression in Equations 5, 6.


(5) $$\begin{array}{l} h_{t}=O_{t}\tanh\left(C_{t}\right) \end{array}$$

(6) $$\begin{array}{l} O_{t}=\sigma \left(W_{o}\cdot \left[h_{t-1},x_{t}\right]+b_{o}\right) \end{array}$$

Where *W*_*o*_ and *b*_*o*_ are the weighted matrices of the output gate and LSTM bias, respectively.

## 3 Materials and methods

With the rapid adoption of remote healthcare services, the risk of cyberattacks and data tampering has increased significantly. The main endeavor of this research is to detect and analyze hidden embeddings in medical images for secure medical data transmission. By continuously analyzing incoming medical images using AI-driven image steganalysis, data security and privacy risks can be minimized. In our proposed architecture, LSTMs were fused within the CNN by replacing the fully connected layers. The idea was to capture and rank the correlation between different stego-noises and to reduce the number of trainable parameters for time efficient classification.

### 3.1 Pre-processing BOSSBase 1.01 and BOWS 2 databases

For the experiments, Break Our Steganographic System (BOSSBase 1.01) (34) and Break Our Watermarking System (BOWS 2) (35) databases were used. Each database has 10,000 cover images in a Portable Gray Map (PGM) format. The data were prepared by resizing all images to 256 × 256 pixels (36). Then, a corresponding steganographic image for each cover image was generated using with payloads of 0.4 bits per pixel (bpp). In the next stage, the data were partitioned to training, validation, and testing sets. 4,000 images were used pairs for training, 1,000 for validation, and 5,000 for testing purposes. Both datasets were merged to generate a database of 20,000 images in which split 14,000 images were used for training (10,000 BOWS 2 + 4,000 BOSSBase 1.01), 1,000 pairs for validation (BOSSBase 1.01), and 5,000 for testing (BOSSBase 1.01).

### 3.2 Pre-processing ALASKA2 image steganalysis database

ALASKA2 dataset was chosen due to its massive size and heterogeneous nature for an in-depth validation of our proposed steganalysis algorithm. In this dataset, steganography algorithms transform data with an unknown payload. All the images were resized to 256 × 256 pixels and compressed with JPEG quality factors of 95, 90, and 75. This database is available on Kaggle platform (37). ALASKA2 database includes 7,500 pairs of images in JPEG format (cover and stego) which were randomly shuffled before partition. We prepared the ALASKA2 database by portioning split 6,000 pairs for training, 1500 pairs for validation, and 7,500 pairs were randomly chosen testing purposes. Furthermore, we prepared another ALASKA2 dataset by using all images via three steganographic algorithms. This database was partitioned in which 9,000 pairs were used for training, 2,250 pairs for validation, and 11,250 pairs for validations.

### 3.3 Proposed LSTM fused CNN architecture

Initially, we establish the effectiveness of LSTM for steganalysis in securing telemedicine communications and then integrate it into a CNN architecture to enhance both detection accuracy and processing efficiency. Given the critical need for real-time threat detection in remote healthcare, we provide a detailed analysis and comparison with state-of-the-art architectures to assess our model's capability. To simulate real-world security threats in telemedicine, we embedded noise in cover images using five steganographic algorithms. Two of them are spatial steganographic algorithms: S-UNIWARD (38) and WOW (39) with 0.4 bpp payloads. The other three are transform steganographic algorithms: JMiPOD (40), JUNIWARD (38), and UERD (41). Our implementation ensures robust steganalysis for secure medical image transmission.

**Figure 3:**
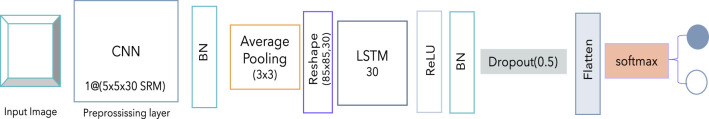
Schematic illustration of LSTM for feature representations and classification.

Our initial approach investigates the applicability of LSTM in image steganalysis and is presented in Figure 3. It starts with an input image, which is first passed through a preprocessing layer using a convolutional neural network (CNN) filter of dimensions (5 × 5 × 30), indicating the use of 30 SRM (Spatial Rich Model) filters for extracting high-frequency residuals. This is followed by batch normalization (BN) to stabilize and accelerate training. Next, average pooling with a 3 × 3 kernel is applied to reduce spatial dimensions while preserving critical features. This is then reshaped into a sequence format (65 × 30), which is suitable for temporal modeling via LSTM. After reshaping, the feature map is fed into an LSTM layer with 30 units as illustrated in Figure 3. The output of LSTM is passed through a ReLU activation to introduce non-linearity, followed by another batch normalization to standardize feature distributions. A dropout layer with a rate of 0.5 is included to prevent overfitting by randomly deactivating neurons during the training. The resulting features are flattened into a one-dimensional vector and are further passed through a Softmax classifier. This architecture combines the spatial feature extraction capability of CNNs with the sequential modeling strength of LSTMs, making it particularly robust for detecting subtle patterns in stego and manipulated images.

**Figure 4:**
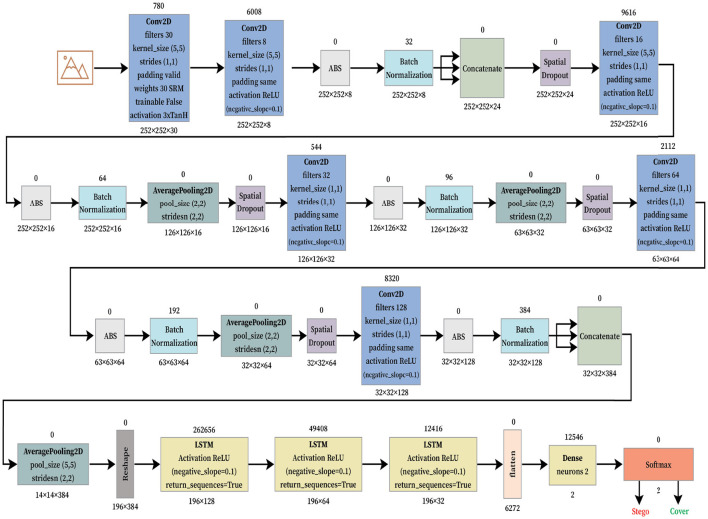
Proposed LSTM fused Xu-Net neural network architecture for secured telemedicine.

After the initial proof of concept regarding LSTM architecture for steganalysis, we fused LSTM as a classifier into the CNN architecture by replacing its three fully connected layers which is presented in Figure 4. The model begins with a convolutional preprocessing layer using fixed SRM filters, which are effective in extracting the noise residuals from the images. These initial outputs are passed through several convolutional blocks, each containing Conv2D layers, batch normalization, and spatial dropout. It is further followed by average pooling to reduce spatial dimensions while maintaining the important feature structures. The model uses concatenation operations to merge different channels for a multi-level residual learning. After the hierarchical CNN feature extraction, the architecture transitions into a temporal modeling phase using LSTM layers. Before entering the LSTM block, features are reshaped and passed through an average pooling 2D layer. The sequence of two LSTM layers allows the model to capture long-range dependencies across spatially transformed image features. The final output from the LSTM is flattened and passed into a dense layer with two neurons, corresponding to a binary classification: Stego and Cover. A softmax layer provides probabilistic outputs for the final decision. This hybrid CNN-LSTM design, coupled with residual modeling, makes the architecture well-suited for subtle signal detection tasks.

**Figure 5:**
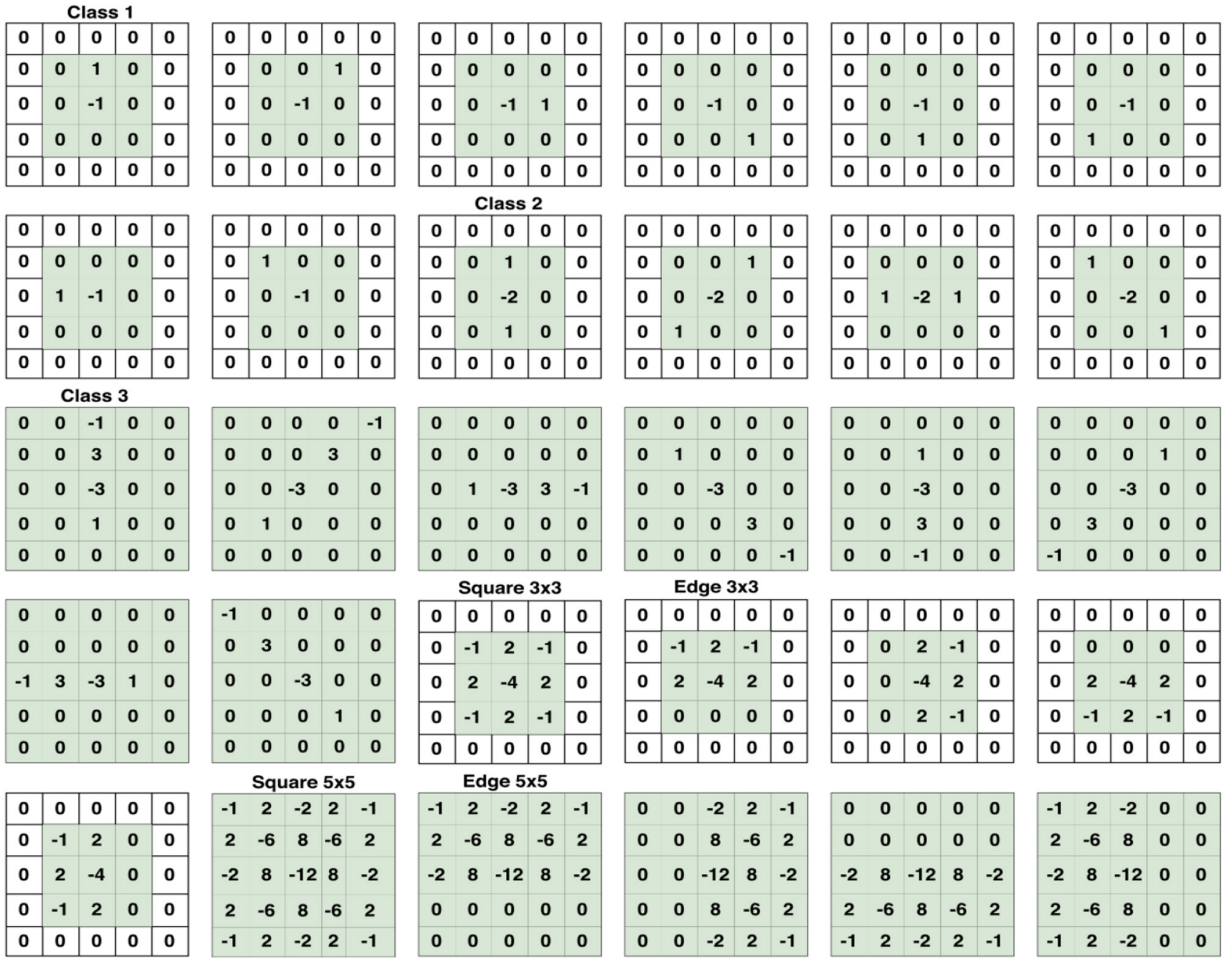
Set of 30 SRM Filters per category which are used in the first convolution, or preprocessing stage. Taken from Reinel et al. (22).

For this experiment, four famous and recent CNNs for image steganalysis were used, which include Xu-Net (24), Ye-Net (15), Yedroudj-Net (18), and Zhu-Net (26). SRM filters were used to improve the ratio of stego- to image-noise signal. Since the stego signal is always embedded in the high-frequency part of an image, we utilized these filters to initialize the kernels of a convolutional layer. A bulk of 30 high-pass filters from the SRM are used in the pre-processing block prior to feature extraction phase as indicated in Figure 5.

Experimental implementations used Python 3.8.1 and TensorFlow 2.2.0. In our model using LSTM only, network was trained for 100 epochs using S-UNWARD steganography with payload 0.4 bpp (BOSSBase 1.01 dataset). The LSTM fused CNN implementations presented in Figure 4 used the Google Colaboratory platform on Tesla P100 PCIe (16 GB) having CUDA Version 10.1 with 32 GB RAM to speed up simulations.

## 4 Results and discussion

### 4.1 Validation of LSTM classifier on BOSSBase 1.01, BOWS 2, and ALASKA2 dataset

**Figure 6:**
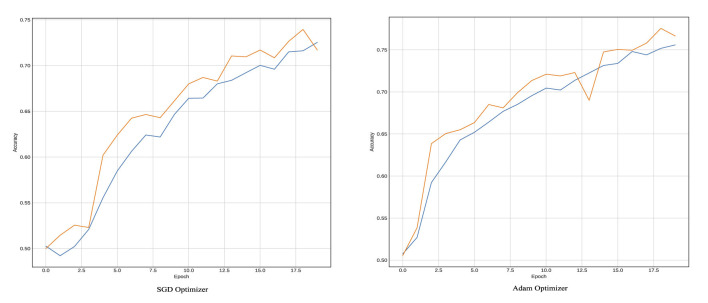
Training plots in terms of accuracy for Yedroudj-Net Model using LSTM as a classifier with BOSSBase 1.01 WOW 0.4 bpp.

To ensure reliable telemedicine, the LSTM classifier was trained for 100 epochs on the BOSSBase 1.01 and BOWS 2 databases and 50 epochs on the ALASKA2 database. A batch size of 64 images was used, with the Stochastic Gradient Descent (SGD) optimizer set at a momentum of 0.95 and an initial learning rate of 0.005. The training curves, illustrating accuracy and learning loss, are presented in Figure 6. Our model incorporates gating mechanisms to regulate gradients, enabling the architecture to retain critical information necessary for detecting hidden threats in transmitted medical images. This ability to learn and preserve information over extended sequences enhances the reliability of telemedicine via secure data transmission.

**Figure 7:**
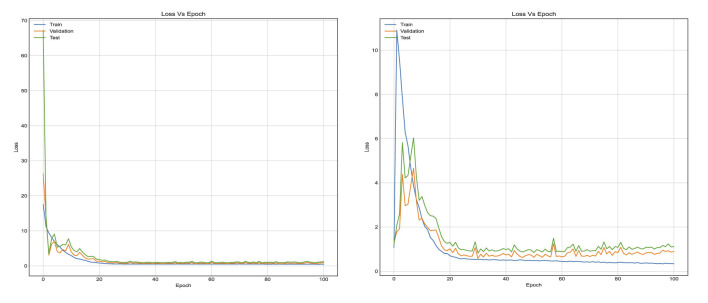
Training curves in terms of learning loss for the first method, when using stacked LSTM and FC layers in the classification stage, respectively. With BOSSBase 1.01 S-UNWARD 0.4 bpp.

Figure 7 reflects the loss function which is binary cross entropy. The results indicate that LSTM model reaches saturation in a time-efficient manner very as the training data hyperparameters were tuned quickly. The gap between validation loss and the training loss using LSTM model is indicative of the fact that LSTM have the ability to adapt to diverse datasets and can generalize to new data. Moreover, the loss value of LSTM model is small and less than that of FC model. The classification accuracy and number of trainable parameters are reported in Table 1 with a fully connected layer and hybrid LSTM for S-UNWARD steganographic algorithm. As presented in Table 1, the fully connected model achieves higher training accuracy (85%) as compared to the LSTM-based model (75%), which suggests that the FC model is better at fitting the training data. However, the similarity in test accuracy between both models indicates that the FC model suffers from overfitting. This is due to specific patterns in the training set that do not generalize well to the unseen data. In contrast, the LSTM model with its inherent regularization via likely promotes better generalization despite its lower training accuracy. This behavior is consistent with the hypothesis that the FC model's capacity to memorize leads to overfitting, while the LSTM model trades some training performance for improved robustness to the unseen data.

**Table 1:** Accuracy percentage and number of trainable parameters of the fist method model, when using FC layer and LSTM layer for the S-UNWARD steganographic algorithm with payload 0.4 bpp using BOSSBase 1.01 database.

**Scenario**	**with LSTM**	**with FC**
Training Acc.	75%	**85%**
Validation Acc.	**76%**	75%
Test Acc.	**67%**	**67%**
# Trainable parameters	**433,592**	434,522

The best performances are shown in bold for each scenario.

Table 2 provides the accuracy and loss results of the CNNs when using either of fully connected (FC) layer or LSTM layer for ALASKA2 databases. Similarly, LSTM classifier outperforms FC on ALASKA2 dataset.

**Table 2:** Accuracy percentage and loss value of the fist method model, when using FC layer and LSTM layer for ALASKA2 database.

**Scenario**	**with LSTM**		**with FC**	
**Database**	**Acc**.	**Loss**	**Acc**.	**loss**
JMiPOD	62%	**.99**	**65%**	1.45
JUNIWARD	60%	**1.00**	**62%**	1.00
UERD	61%	**0.90**	**63%**	0.94
ALASKA2_All	**49%**	**1.00**	46%	1.7

The best performances are shown in bold for each scenario.

### 4.2 Validation of LSTM fused CNN architecture against BOSSBase 1.01, BOWS 2, and ALASKA2 dataset

In our proposed model for secure telemedicine, the training batch size was set to 64 images for Xu-Net, Ye-Net, and Yedroudj-Net, while Zhu-Net utilized a batch size of 32. These mini-batches optimize computational efficiency, ensuring rapid and scalable analysis of medical images in remote healthcare environments. To enhance model stability and accuracy in detecting hidden threats in transmitted medical data, we trained Xu-Net, Ye-Net, and Yedroudj-Net for 150 epochs, while Zhu-Net was trained for 70 epochs. A spatial dropout rate of 0.1 was applied across all layers to prevent overfitting, and batch normalization was configured with a momentum of 0.2, epsilon of 0.001, and renorm momentum of 0.4. The Adam optimizer, with a learning rate of 0.001, beta 1 of 0.9, beta 2 of 0.999, and an epsilon value of 1*e*−08, was employed to ensure efficient convergence. To reinforce security in telemedicine image transmission, all layers were regularized for weights and bias, enabling the model to detect anomalies and steganographic threats in real-time. The accuracy results for both the S-UNWARD and WOW steganographic algorithms, which assess the model's ability to identify hidden data in medical images, are presented in Tables 3, 4.

**Table 3:** Accuracy percentage of the second method models for the S-UNWARD steganographic algorithm with payload 0.4 bpp.

**Dataset**	**BOSSBase 1.01**			**BOSSBase 1.01**+ **BOWS**		
**results**	**Original**	**Strategy**	**With LSTM**	**Original**	**Strategy**	**With LSTM**
Xu-Net	73%	78%	76%	–	82%	81%
Ye-Net	68%	81%	80%	–	83%	81%
Yedroudj-Net	77%	79%	79%	–	84%	82%
Zhu-Net	84.5%	78.6%	80.7%	–	86%	81.3%

**Table 4:** Accuracy percentage of the second method models for the WOW steganographic algorithm with payload 0.4 bpp.

**Dataset**	**BOSSBase 1.01**			**BOSSBase 1.01**+ **BOWS**		
**Results**	**Original**	**Strategy**	**With LSTM**	**Original**	**Strategy**	**With LSTM**
Xu-Net	79%	82%	81%	–	85%	83%
Ye-Net	75%	84%	83%	–	86%	85%
Yedroudj-Net	84%	85%	83%	–	86%	85%
Zhu-Net	88.1%	82.9%	83.5%	–	75%	83.5%

**Figure 8:**
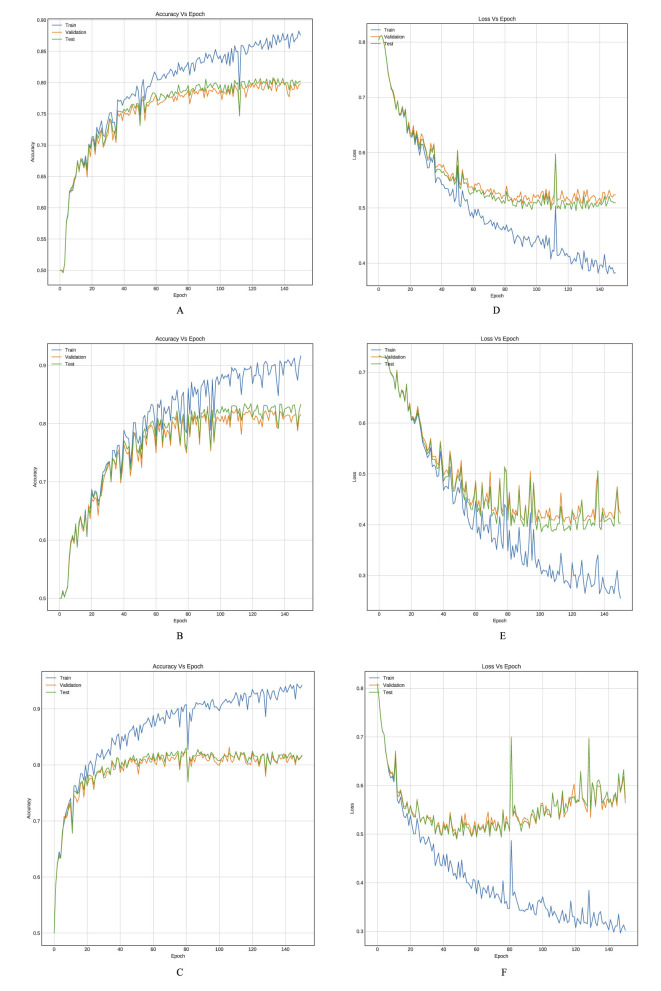
Training curves, **(A–C)** reflect the accuracy, and **(D–F)** reflect the learning loss for Xu-Net based on LSTM, Ye-Net based on LSTM, and Yedroudj-Net based on LSTM, respectively, with BOSSBase 1.01 WOW 0.4 bpp.

**Figure 9:**
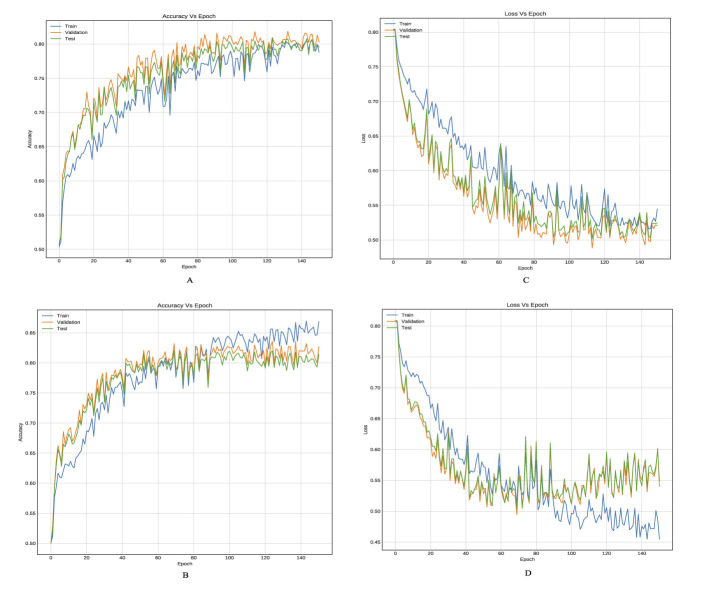
Training curves, **(A, B)** reflect the accuracy, and **(C, D)** reflect the learning loss for Xu-Net based on LSTM, and Yedroudj-Net based on LSTM, respectively, with BOSSBase 1.01 + BOWS 2 S-UNWARD 0.4 bpp.

Tables 3, 4 provide an inter-comparison between the accuracy of our proposed LSTM fused CNN architecture with the reported results (36). We achieved a high agreement between strategy and our model in terms of accuracy. The results highlighted in Tables 3, 4 are extracted from Figures 8, 9.

Trainable parameters refer to those parameters which can be learned and updated during the training cycle and has direct relationship with the computation time. Table 5 presents the number of trainable parameters for each model when applying the strategy reported in Tabares-Soto et al. (36) and when we used our proposed hybrid LSTM model.

**Table 5:** Number of trainable parameters for state-of-the-arts architectures.

**Results**	**Based on FC**		**Based on LSTM**	
**#Trainable parameters**	**Total**	**Classification stage**	**Total**	**Classification stage**
Xu-Net	86,554	59,616	**39,418**	**0**
Ye-Net	**87,562**	22,752	118,570	**0**
Yedroudj-Net	251,110	59,616	**203,974**	**0**
Zhu-Net	275,684	59,616	**265,156**	**0**

The best results are shown in bold for each scenario.

The results presented in Table 5 confirm that our proposed model significantly decreased the number of trainable parameters as compared to leading available models and hence the computational effort required.

## 5 Conclusion

Our proposed architecture proves to be highly effective in capturing complex interrelations among different features, making it a viable choice for steganalysis in telemedicine. Experiments conducted on BOSSBase 1.01, BOWS, and ALASKA2 datasets validate that our model demonstrates strong adaptability and generalization capabilities, which are essential for detecting hidden manipulations in telemedicine imaging systems. The achieved validation loss characteristics further reinforce the robustness of our approach in identifying steganographic threats in medical data transmission. A comparative analysis with leading architectures highlights that our model achieves significant dimensionality reduction in terms of training parameters, making it more efficient without compromising accuracy. This efficiency is critical for real-time telemedicine applications.

However, we acknowledge that the current study does not include validation on real-world clinical datasets or standard medical image formats such as DICOM. Addressing this limitation forms a key part of our future work, where we aim to evaluate the model's performance on actual clinical imaging data to strengthen its practical applicability in telemedicine settings. By continuing to refine and expand our approach, we can contribute to a more secure and reliable telemedicine ecosystem.

## Data Availability

Publicly available datasets were analyzed in this study. The code is available on GitHub: https://github.com/DrDoaaSh/phd-code.git. The data set used to reproduce the results can be downloaded from this link: doi: 10.5281/zenodo.4884116 or from this link https://drive.google.com/drive/folders/18KaJnn432D89WJarNY5NCTAxZB2Z3nw7?usp=drive_link.
